# Experimental Study of LoRa Transmission over Seawater

**DOI:** 10.3390/s18092853

**Published:** 2018-08-29

**Authors:** Nikola Jovalekic, Vujo Drndarevic, Ermanno Pietrosemoli, Iain Darby, Marco Zennaro

**Affiliations:** 1 Department of Electronics, School of Electrical Engineering, University of Belgrade, Bulevar Kralja Aleksandra 73, 11060 Belgrade, Serbia; vujo@etf.rs; 2 Telecommunications/ICT4D Lab, ICTP, Str. Costiera 11, 34151 Trieste, Italy; ermanno@ictp.it (E.P.); mzennaro@ictp.it (M.Z.); 3 IAEA Nuclear Science and Instrumentation Laboratory, IAEA Laboratories Seibersdorf A-2444, 2444 Seibersdorf, Austria; I.Darby@iaea.org

**Keywords:** LoRa, IoT, LPWAN, sea, transmission

## Abstract

Low Power Wide Area Networks (LPWANs) are gaining attention in both academia and industry by offering the possibility of connecting a large number of nodes over extended distances. LoRa is one of the technologies used as a physical layer in such networks. This paper investigates the LoRa links over seawater in two typical scenarios: clear Line-of-Sight (LOS) and obstructed path in two different Industrial, Scientific and Medical (ISM) radio bands: 868MHz and 434MHz. We used three different LoRa devices in the experiments: the Own Developed LoRa Transceiver (ODT) and two commercial transceivers. Firstly we investigated transceivers’ Receive Signal Strength Indicator (RSSI) and Signal-to-Noise (SNR) measurement chain linearity and provided correction factors for RSSI to correlate it with actual signal levels received at transceivers’ inputs. Next, we carried out field experiments for three different LoRa Spreading Factors, SF∈[7,10,12], within a bandwidth of BW=125kHz and Coding Rate CR=4/6. The experiments showed that LoRa links are fully feasible over seawater at distances at least 22km long, using only low-cost off-the-shelf rubber duck antennas in LOS path condition in both ISM bands. In addition, we showed that LoRa links can be established over 28km obstructed LOS oversea path in ISM 434MHz band, but using costly, higher gain antennas. Furthermore, the laboratory experiments revealed that RSSI is linear in a wide range, up to −50dBm, whereas the SNR measurement chain goes into saturation for Received Signal Strength (RSS) values higher than −100dBm. These findings enabled accurate interpretation of the results obtained in field experiments.

## 1. Introduction

Deployment of sensors in the marine environment is a demanding task and among many implementation-related challenges, it requires reliable and low power wireless communication links between the nodes and base stations or gateways. Usually, in coastal regions, technologies like WiFi or Zigbee are used, provided that there is LOS between the access point and the node that contains the sensors. When nodes are deployed in remote sea locations, two widely adopted technologies are exploited: Global System for Mobile Communications (GSM) or data transfer over satellite constellation, such as Iridium [1].

A number of scientific works addressed the problem of data transfer from the measurement node using the technologies mentioned. For example, Kazdaridis et al. [2] successfully demonstrated the use of sub-gigahertz transceiver to transfer measurement data from a buoy located 2.5km from the seashore, using narrow band type of modulation. Boydstun et al. [3] carried out data transfer using WiFi over a distance of approximately 300m, while Pozzebon et al. [4] demonstrated successful communication in coastal region combining both ZigBee and GSM. The work presented by Trasvina-Moreno [5] showed successful communication with measurement node using the Iridium satellite constellation.

As can be concluded, to be able to cover different sensor deployment scenarios, various technologies have to be utilized. For example, ZigBee and WiFi are adequate technologies for deployments of up to several hundred meters from the coast, which limits their usage in remote areas. On the other hand, GSM technology can be used in remote areas, up to 35km, provided that there is general network coverage of the area. However, cost of data transfer has to be taken into account since GSM technology utilizes the licensed part of the spectrum. In addition, all three technologies offer data rates which are significantly higher than it is really required for most sensor deployments. The power consumption of devices based on these technologies is significant since complex transmission protocols have to be processed per byte of the payload. Finally, deployments using satellite data transfer are rarely exploited due to the cost of the data transfer and implementation of the measurement node.

LoRa, as one of the LPWAN underlying technologies, offers several advantages in deployments over seawater. First of all, it enables better range in comparison to all three technologies due to its sensitivity which goes down to −148dBm [6]. Since it utilizes the unlicensed part of the spectrum, there is no cost of data transfer. In addition, due to low data rate and small transmission protocol overhead per byte of payload, power consumption is very low, offering extended battery-powered operating time.

However, there are fewer works that tackle data transmission over seawater utilizing LoRa technology. Petajajarvi et al. [7] carried out several experiments in different scenarios, including experiments over seawater. Their setup consisted of a commercial node, which was sending packets periodically with a sequence number, node status, and coordinates obtained by GPS. Packets were being received by the gateway installed on the shore on a fixed position. The node was installed on a boat which was sailing away from the shore, transmitting packets in the 868MHz band, randomly switching the channels within the band. The output power was set to 14dBm, and the inter-packet delay was set to be in accordance with ETSI [8] regulation for duty cycling. The results were presented in a grossly aggregated metrics, showing a maximum communication distance of 30km with packet loss of 32% on 3688 packets sent. In addition, they derived a path loss model for each experimental scenario.

Lingling et al. [9] deployed LoRa transceiver in a sailing monitoring system, using it to transmit the sailboat’s parameters to a gateway in the 434MHz band, utilizing a bandwidth of BW=125kHz and a spreading factor SF=7. The experiment results showed a maximum distance of 3467m with packet loss of 63.26% on 1195 packets sent. The results were also presented in a grossly aggregated metrics fashion.

The conclusions drawn from these experiments are restricted for several reasons. In both works, a relatively small number of packets was sent, with a maximum of 3688 packets. To assess link behavior and provide enough data for statistical processing, usual engineering practice recommends at least 10,000 packets to be transmitted. Next, data are presented cumulatively, in the form of packet loss, therefore no insight into link behavior over time can be gained. In addition, the selection of only one set of LoRa parameters deployed in the experiments cannot provide general insights in the link behavior because parameters are either selected to enable maximum sensitivity of the transceivers, as in [7] (SF=12, BW=125kHz), or to take into account trade-off between power consumption, data rate and transmission delay as in [9] (SF=7, BW=125kHz). In addition, in both works, only one frequency band is investigated, namely 868MHz in [7] and 433MHz in [9], hence advantages of using one over another frequency band cannot be quantified. Moreover, only one pair of commercial LoRa transceivers were used in the experiments; this can limit the insight in the technology capabilities, especially if we take into account the fact that commercial transceivers are usually not optimized in terms of sensitivity [10]. This is due to different trade-offs in the design which are necessary to be done in order to build general purpose transceiver. As a further matter, while in [7] RSS is logged, in [9] there is no information provided at all; additionally, both works lack data for Signal-to-Noise Ratio (SNR) for received packets, which limits gaining a deeper insight into technology and links’ behavior. Besides, scaling factors for RSS indicator should be experimentally measured, and linearity and saturation region of SNR measurement chain investigated to provide an accurate interpretation of logged quantities.

Finally, these two groups of experiments cover, in some aspects, one of two important deployment scenarios—when nodes are mobile, maintaining LOS, and are mounted near the seawater surface. However, there is another big group of potential deployments, which assumes that nodes are fixed and mounted well above the seawater surface. This is the case, for example, in island countries where there is a need to connect, e.g., environmental monitoring stations on different islands, or to implement early warning systems for detection of volcano eruptions, etc. The results from previous works are not fully applicable in these scenarios also because the nodes used in the experiments were deployed very close to seawater surface, which can alter the antenna radiation pattern and its resonant frequency and, consequently, degrade packet reception rate. Contrariwise, when nodes are fixed and deployed on the mainland, these effects are not dominant, and a higher packet reception rate could be possibly obtained.

In our research, we aimed to investigate LoRa links over seawater in two characteristic scenarios: LOS and obstructed LOS at two different frequencies simultaneously, namely 868MHz, and 434MHz. In order to do so, we addressed all the potential limitations we found in currently available works. We increased significantly the number of packets sent by sending 20,000 packets in each experiment, and carried out data analysis in the time domain, exploring short and long-term variabilities in terms of RSS, SNR, and Packet Reception Rate (PRR); we also presented cumulative analysis in the form of histograms and packet loss. In addition, we explored different LoRa parameters, covering all characteristic cases: (1) minimum sensitivity of the transceiver by setting SF=7 and BW=125kHz; (2) mid-range value as a trade-off between sensitivity and data rate by setting SF=10 and BW=125kHz; and (3) maximum sensitivity by setting SF=12 and BW=125kHz. To make experimental data more reliable, we deployed three different transceivers on the receive side, where one was own-developed and optimized in terms of sensitivity [10], and two were commercial transceivers. Finally, to provide an accurate interpretation of experimental data, we performed laboratory measurements and provided scaling factors for RSS indicators of all three transceivers, and determined linear and saturation regions of operation for SNR measurement chain of each transceiver used in the experiments.

The results of this study show that LoRa links over seawater are fully feasible in LOS propagation condition at both frequencies, namely 868MHz and 434MHz up to at least 22km. The link margin, which yields more than 15dB in each experimental scenario suggests that the link length can be even longer. Furthermore, due to use of the characteristic sets of LoRa parameters (lowest, mid-range and highest value for SFs, and lowest for BW standardized by LoRaWAN) it can be concluded that links are feasible for virtually any set of LoRa parameters. Deployment of low-cost off-the-shelf rubber duck antennas on both transmitting and receiving side extends the usability of the experimental results also to commercial deployment arena. Finally, this study shows that links in obstructed LOS propagation mode are only possible at 434MHz using costly and bulky antennas on both sides.

The paper is laid out as follows: Section 2 briefly introduces LoRa technology, while in Section 3 materials, experimental setup, and RSSI correction factors are given. In addition, SNR measurement chain linearity is investigated. Section 4 presents and analyzes experimental results, while in Section 5 conclusion remarks are given.

## 2. LoRa Technology

**Figure 1:**
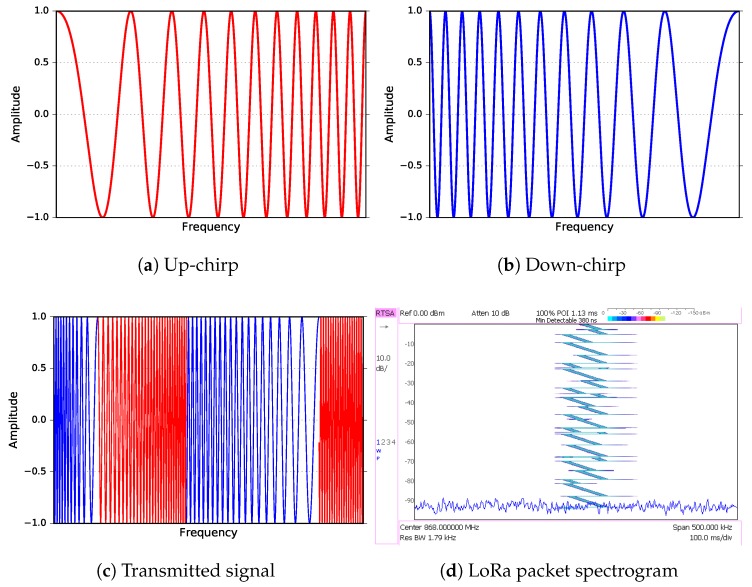
LoRa physical layer. Signals used to convey information: (**a**) up-chirp and (**b**) down-chirp; (**c**) transmitted signal composed of two up-chirps and two down-chirps; (**d**) LoRa packet spectrogram captured by real-time spectrum analyzer.

LoRa [6,11] is proprietary modulation based on spread spectrum techniques developed by Semtech. In this type of modulation, the bandwidth spreading is done using “chirps”, i.e., pulses of linearly increasing (or decreasing) frequency, spanning the whole bandwidth allocated for transmission. An example of such chirps is shown in Figure 1.

Data to be transmitted is encoded in abrupt changes from up-chirp to down-chirp or vice-versa, maintaining a constant envelope of the modulated signal [12], which simplifies the design of the amplifiers since there is no need for the complex and expensive ones required by other types of modulation [13]. At the receiver side, the same $F_{c}$ tone is used to down-convert the signal to baseband, where essentially the reverse of the operations performed in transmission is undertaken to recover the original data affected by the inevitable noise.

Demodulation is accomplished in two steps: (1) multiplying the received signal by the conjugate of each of the two chirps used in transmission, which turns each modulated symbol into regions of constant frequency; and (2) performing a Fast Fourier Transform (FFT) in each region. The peak of the FFT represents the value of the received symbol, easily distinguishable even in the presence of considerable noise or interference [14].

There are three LoRa parameters that enable the designer of the LoRa link to balance between bit rate and robustness of the transmission: Bandwidth (BW), Spreading Factor (SF), and Coding Rate (CR). The bandwidths used in LoRa links can take BW∈[125,250,500]kHz, while spreading factors can be SF∈[6,12]. Coding rate offers the possibility to add up to four redundant bits for error handling after every 4-bit chunk of payload transmitted, making the transmission more resistant to interference bursts.

**Figure 2:**
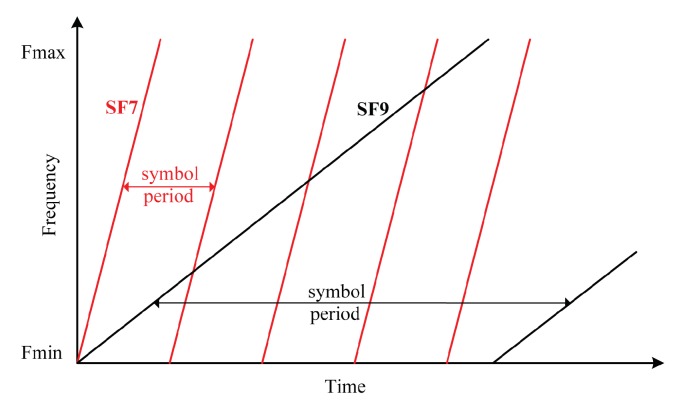
Trade-off between symbol period and spreading factor.

We can opt for higher sensitivity by choosing a higher spreading factor, or for a faster bit rate by selecting lower spreading factor. Higher spreading factors result in longer symbol periods and longer packet air times, whereas a lower spreading factor yields shorter transmission times. These trade-offs are illustrated in Figure 2.

Finally, LoRa technology offers two advantages over other technologies used in LPWANs. Since the modulated signal is continuously changing in frequency, it is more tolerant to clock inaccuracies and also to Doppler shifts caused by movements between the transmitter and the receiver [15]. Additionally, the main modulation mechanism i.e., abrupt change of the frequency, facilitates reception of very weak LoRa signals, even below the noise floor of the transceiver.

## 3. Materials, Experimental Setup and Methods

Numerous experiments were carried out in both LOS and obstructed paths for a variety of LoRa parameters and in two different ISM frequency bands. The layout of each experiment was identical; namely, two different ODTs were sending packets simultaneously at 868MHz and 434MHz, while three different transceivers were receiving packets at 868MHz and an additional one at 434MHz. In the 868MHz band, ODT [10], Libelium [16], and Uputronics [17] were receiving packets in parallel, while another one ODT was receiving packets at 434MHz. Commercial transceivers were selected based on their cost and prevalence in different deployments. Libelium is a costly solution, whereas Uputronics is a low-cost solution; both solutions are widely adopted in industry.

### 3.1. Brief Description of the Transceivers Used in the Experiments

The ODT utilizes the SX1276 [18,19] LoRa modem from Semtech. The modem can operate in several frequency bands using two different RF ports: one for frequencies up to 525MHz, and one for 525–1020 MHz. The operating frequency band is controlled by the register settings over the following values: 169,434,470,490,868MHz and 915MHz, but changing the frequency band requires also changing the corresponding impedance matching in the RF front end to prevent reflections that degrade the performance. The ODT was fitted with a matching network for two ISM bands: 868MHz and 434MHz, providing 14dBm of output power on the high-frequency port and 20dBm on the low-frequency one. The transceiver is capable of using seven different spreading factors, SF∈[6,12], within four different bandwidths: 62.5,125,250, and 500kHz.

**Figure 3:**
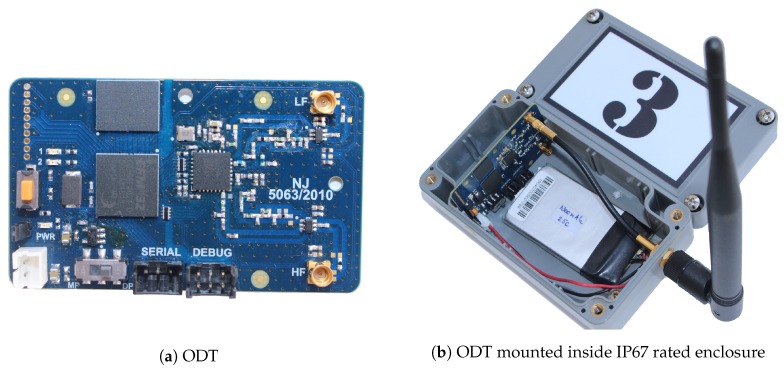
Physical appearance of: (**a**) ODT, (**b**) device deployed in the field experiments.

Besides the LoRa modem, the ODT features the Energy Micro EFM32GG990F1024 [20] microcontroller and 32mB of NAND flash memory from Micron, NAND256W3A2BZA6E [21] for logging purposes. There is also one expansion port for connecting different types of sensors, two LEDs for signaling, and a push button. The ODT is depicted in Figure 3a, while the complete device used in the field experiments is shown in Figure 3b.

The Libelium device uses the Semtech SX1272 [22] LoRa modem, and supports only one frequency band, 860–1020 MHz, offering output power up to 20dBm. It can use spreading factors in the range of SF∈[6,12], within three different bandwidths: 125,250, and 500kHz. This transceiver is mounted on a Seeeduino Stalker v2.3 carrier board [23].

The Uputronics commercial transceiver uses the RFM95 LoRa module from HopeRF [24], which has identical characteristics to those of the SX1276, as determined by comparing the corresponding datasheets. As with the Libelium transceiver, this also requires a host system for its operation and is therefore mounted as a shield on a Raspberry Pi3 [25].

Omnidirectional antennas were used in all experiments: for the LOS condition, low-cost off-the-shelf rubber duck antennas were deployed in both frequency bands. The antennas have a gain of 4.5dBi and 0.7dBi for 868MHz and 434MHz, respectively. In the obstructed LOS path scenario, three element collinear dipoles were deployed as well, providing gains of 8.5dBi and 6.5dBi for 868MHz and 434MHz band, respectively.

### 3.2. Experimental Locations and Setup

**Figure 4:**
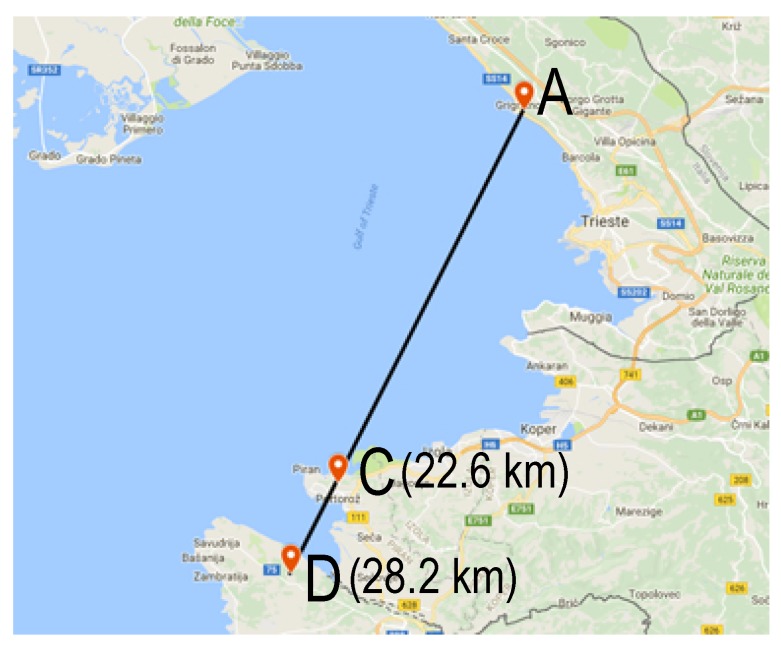
Map showing line-of-sight trajectory (A–C) and obstructed trajectory (A–D).

The experimental locations were found using BotRf [26], a software tool for wireless link planning that uses the Longley-Rice model to estimate path loss on a given trajectory using digital elevation maps. The locations were selected based on maximum attainable LOS and obstructed LOS link distance in Gulf of Trieste. The process of finding suitable test locations was carried out through several iterations, considering also the physical suitability for equipment deployment. Geographical details of the sites that fulfill such requirements, and which were used in the experiments are given in Table 1, while Figure 4 provides details on the map.

**Figure 5:**
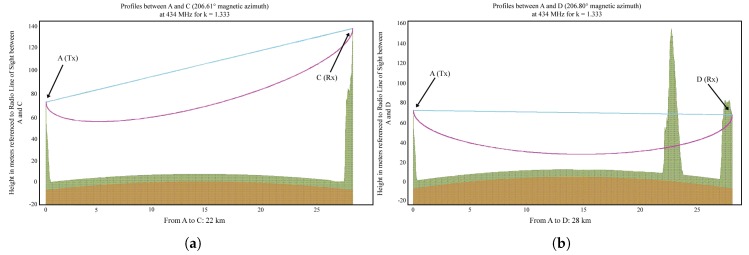
Terrain profiles for: (**a**) Line-of-Sight path over sea. (**b**) Obstructed path over sea. Brown area indicates the curvature of the earth, modified by the refraction index; Green area is the terrain profile as seen by the radio wave and magenta line corresponds to 60% of the first Fresnel zone at 434MHz; and blue line is the optical line of sight.

The simulation results obtained using BotRf are summarized in Table 2, while the terrain profiles are shown in Figure 5.

The transmitters were deployed at Site A during all experiments, while the receiving nodes were deployed at Site C in the first group of experiments (LOS) and at Site D (obstructed LOS) in the second group of experiments.

### 3.3. Traffic Setup

In all experiments, the transmitters were configured to send packets consisting of an 8 byte preamble, 9 byte payload and CRC. The payload of each packet was populated with the packet’s unique identification number (ID) which was consecutively incremented in each sending cycle starting from the value ID=1. An inter-packet sending delay of $T_{d}=200\;$ms was set to enable also the investigation of short-term variabilities in reception quality. However, it should be noted that this sending rate violates the European constraints of maximum duty-cycle [8], and should not be embraced in commercial solutions. On the receive side, time stamp, packet ID, RSS, and SNR were logged.

Experiments were carried out within a bandwidth of BW=125kHz and a coding rate of 4/6, while the spreading factor was varied, SF∈[7,10,12]. The bandwidth was fixed at 125kHz since it is the value standardized by the LoRaWAN specification, while the coding rate of 4/6 was chosen as a mid-value among those supported by the LoRa modem.

It is worth remembering that different LoRa parameters yield different packet air times. Since 20,000 packets were sent in each experiment, the duration of the experiments was different. Table 3 summarizes packet air time, equivalent bit rate, theoretical sensitivity and experiments’ duration for the LoRa parameters used in the measurements.

### 3.4. RSSI Correction Factors and SNR Measurement Chain Linearity

LoRa ICs are equipped with RSS indicators and SNR measurement capabilities. Measured values are stored in the corresponding registers and can be read via the SPI bus by the host processor. For the proper interpretation of the experimental results, it is necessary to assess RSSI accuracy and determine the saturation threshold for the SNR measurement chain. However, until now, there is no work which fully addresses this issue. For example, Gaelens et al. [27] found the SNR measurement chain saturation threshold for the transceiver used in their experiments, but without finding RSSI correction factors.

RSSI correction factors imply correlation of actual received power at the IC’s RF pin and the value available in the corresponding transceiver’s register. The measurement offset may be a consequence of the transceiver’s RF front end attenuation and/or the inherent IC production tolerances.

**Figure 6:**
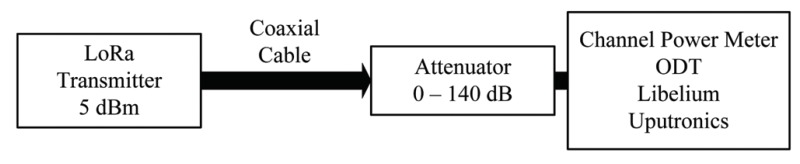
Measurement setup for RSSI correction factors and SNR measurement chain linearity.

To find RSSI correction factors and investigate SNR measurement chain linearity, we arranged the setup shown in Figure 6.

**Figure 7:**
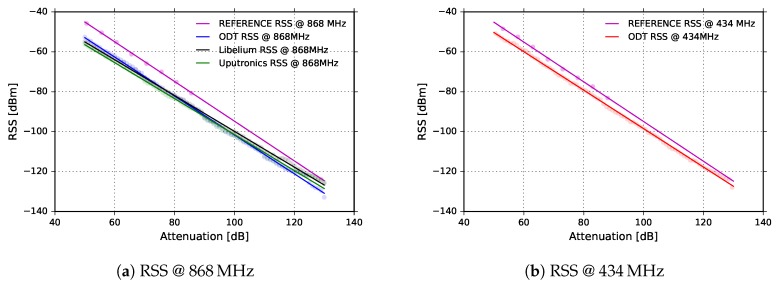
Received signal strength vs. channel attenuation for reference RSS and (**a**) ODT (868MHz), Libelium (868MHz) and Uputronics (868MHz), (**b**) ODT (434MHz).

An ODT was used as a transmitter set to 5dBm of output power. The output was connected through a coaxial cable and a variable attenuator to the channel power meter (Keysight FieldFox N9915A handheld microwave analyzer). We performed channel power measurements on LoRa signals at different attenuations and for both frequencies of interests. The results are presented in Figure 7a,b in magenta color for both 868MHz and 434MHz. In that way, we obtained reference RSS values for both frequencies.

We then repeated the same measurements replacing the channel power meter with each of the LoRa transceivers used in the field experiments, recording RSS and SNR for both frequencies of interest. The results are presented in the same plots.

Applying a least squares error polynomial fit through the measured points we obtained the following equations:

(1) $$Reference_{434\mathrm{MHz}}=-0.9948\times att+4.556$$

(2) $$Reference_{868\mathrm{MHz}}=-0.9938\times att+4.628$$

(3) $$ODT_{434\mathrm{MHz}}=-0.9626\times att-2.205$$

(4) $$ODT_{868\mathrm{MHz}}=-0.9743\times att-4.194$$

(5) $$Uputronics_{868\mathrm{MHz}}=-0.9007\times att-11.41$$

(6) $$Libelium_{868\mathrm{MHz}}=-0.8962\times att-10.22$$

The obtained equations were used to correct the values reported by corresponding transceivers. As can be seen in Figure 7a,b, RSS reported by the transceivers is linear in a wide range, although there is a constant offset between the power measured with the power meter and the corresponding values read in the devices’ registers. The difference is about 6.7dB at 434MHz and about 8.8dB at 868MHz for ODT. The commercial devices exhibit different offsets due to different RF front-end attenuations: 16dB for the Uputronics and 14.8dB for the Libelium. It can be inferred that the devices are self-coherent but the absolute values reported by the transceivers significantly differ from absolute values measured with the power meter.

**Figure 8:**
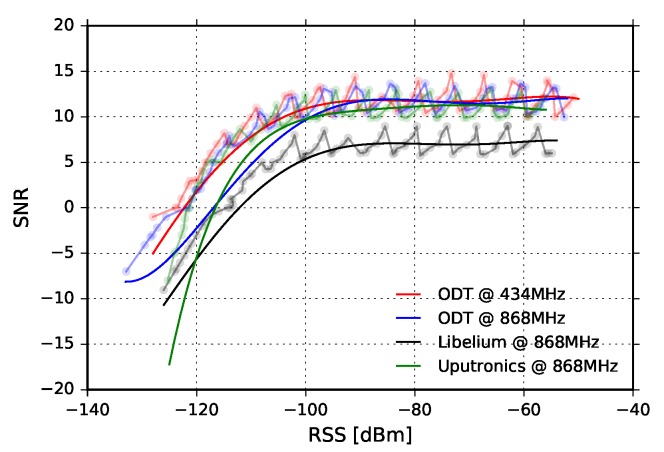
Signal-to-Noise ratio vs. Received signal strength for ODT (434MHz), ODT (868MHz), Libelium (868MHz) and Uputronics (868MHz).

The linearity of the SNR reported by the transceivers is also experimentally verified for all transceivers used in the experiments. As can be noticed in the Figure 8, SNR measurement chain enters saturation when the RSS approaches −100dBm for all three transceivers.

### 3.5. Weather Conditions

The experiments performed in LOS scenario were conducted during sunny days with an average temperature of $28{\;}^{\circ }$C. On the other hand, measurements done in obstructed LOS test case were carried out during winter on clear days with an average temperature of $4{\;}^{\circ }$C. The amplitude of sea waves in both cases did not exceed 45cm.

## 4. Experimental Results and Analysis

The experimental results with analysis are organized into two groups, based on the propagation condition; inside the groups, data is further divided based on the transceivers’ operating frequency. At the end of the section, a discussion and result generalization in different aspects is given, as well as potential application areas.

During each experiment, RSS, SNR, and PRR were logged. The results are presented both in time domain and also cumulatively, using histograms. The plots in time domain show current values represented by dots, while their moving average values calculated on the 1-min window are shown with solid lines.

The distribution of the moving average values, as well as the difference between current and moving average values, are presented in the form of histograms. In that way, we aimed to improve link analysis and potentially separate interference caused by reflections and terrain shielding from radio-interference.

### 4.1. LOS Scenario

These experiments were carried out within the fixed bandwidth, BW=125kHz, and using constant coding rate, CR=4/6, while the spreading factor was varied, SF∈[7,10,12]. The measurements were performed in the 868MHz and 434MHz bands concurrently.

#### 4.1.1. ISM 868MHz

**Figure 9:**
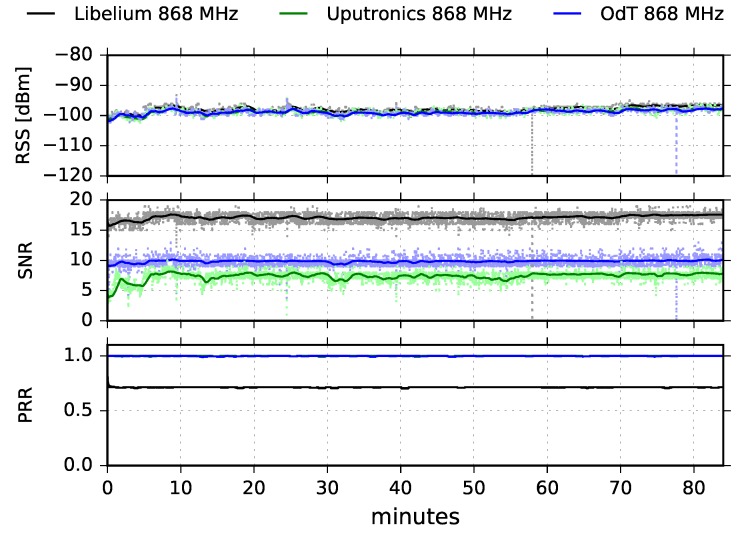
RSS, SNR, and PRR for ODT, Libelium, and Uputronics at 868MHz in LOS trajectory for BW=125kHz, SF=7 and CR=4/6.

Figure 9 shows the results obtained in LOS scenario for ODT, Libelium and Uputronics transceivers, operating at 868MHz with following parameters: BW=125kHz, SF=7, and CR=4/6.

We observe that the RSS is almost constant during the whole experiment and that it is nearly equal for all three transceivers. This implies that multipath propagation effects were not dominant during the experiment and that the main component of the transmitted signal was the one which traveled in direct LOS path. In addition, there can be seen two short dips in 58 and 78 min of the test. They are caused by short radio-interference bursts, but they did not produce a drop in the packet reception rate. On the other hand, the SNR follows the fluctuation trend of RSS for all three transceivers. By inspecting the SNR values of different transceivers, and rescaling Libelium SNR values using Figure 8, we observe that ODT has the highest SNR, and consequently the lowest noise floor; this implies the highest sensitivity of the ODT transceiver.

The PRR for ODT and Uputronics is constantly high, with negligible packet loss; Libelium has a constant packet loss, yielding PL=28.68% which is high and unexpected since the values of RSS and SNR are deeply inside the link margin and there is negligible interference. The additional tests are required in order to investigate the cause of the high packet loss.

**Figure 10:**
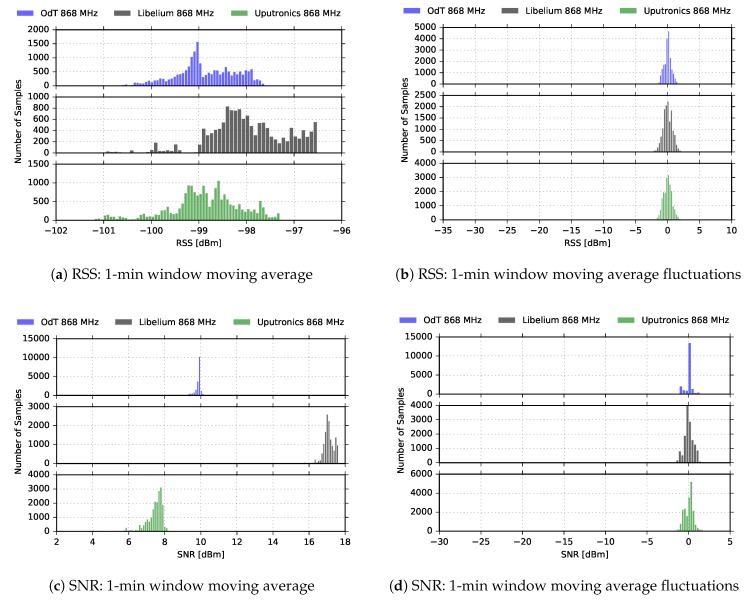
RSS and SNR histograms for ODT, Uputronics and Libelium: f=868MHz, BW=125kHz, SF=7, CR=4/6, LOS.

Figure 10 shows RSS and SNR cumulative results on the entire measurement interval for the ODT, Uputronics and Libelium transceivers at 868MHz, for the same LoRa parameters.

It can be noticed that distribution of RSS values does not indicate multipath propagation effects and fading since Rice distribution cannot be observed. Rice distribution of RSS amplitudes is characteristic when there is a multipath propagation effect in the channel and one component is dominant, usually, the one that travels in LOS path [28]. However, the distribution of the RSS amplitudes has to be interpreted cautiously since the RSS indicator measures the received power of the whole packet and cannot measure the RSS value of a single multipath component. The difference between the 1-min window moving average values and current values exhibits the Gaussian distribution. This indicates that the time window for moving average calculations has been adequately selected since that difference represents the RSS fluctuations, which can be treated as a kind of “noise”.

**Figure 11:**
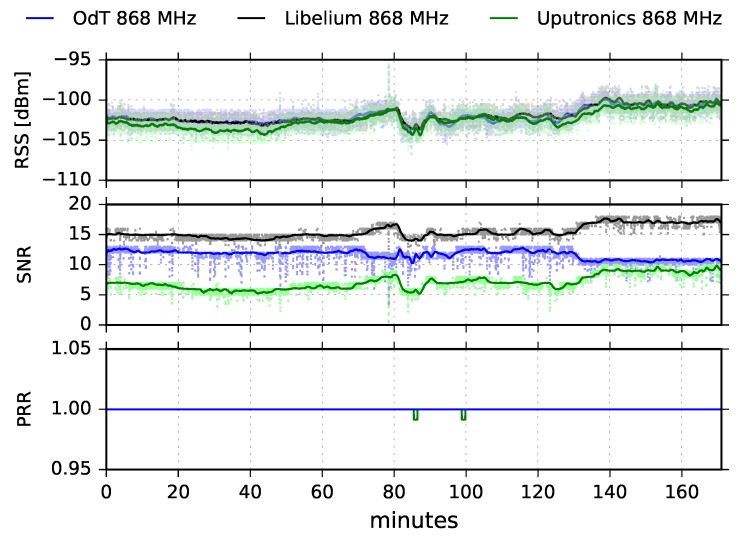
RSS, SNR, and PRR for ODT, Libelium, and Uputronics at 868MHz in LOS channel conditions for BW=125kHz, SF=10, and CR=4/6.

Figure 11 shows results for the ODT, Uputronics, and Libelium transceiver at 868MHz, in LOS scenario, with the following parameters: BW=125kHz, SF=10, CR=4/6.

It can be noticed that in this experiment, RSS of all three transceivers fluctuated. Since these fluctuations are relatively slow, they can be attributed to multipath propagation effects. In the first 70 min of the experiment multipath was not pronounced, however, between 70 and 140 min of the experiment fluctuations of 5dB are recorded. Since the experiment was carried out for LOS path (60% of the First Fresnel zone was clear), multipath was a consequence of meteorological conditions which could cause refraction and consequently reflection from the sea surface by forming air masses with different refraction indexes. However, the multipath did not cause any significant packet loss, Uputronics experienced negligible packet loss of PL=0.01%.

We also observe that SNR recorded by Libelium and Uputronics follows the fluctuation trend of corresponding RSS values. On the other hand, the ODT exhibits counterintuitive behavior after 130 min of the experiment since with the increasing of the signal level at the input, SNR decreases. Examining Figure 8 and taking into account the tolerances of components, this can be explained by saturated SNR measurement chain, which shows the “saw-tooth” oscillating behavior for the input signal levels higher than −100dBm.

**Figure 12:**
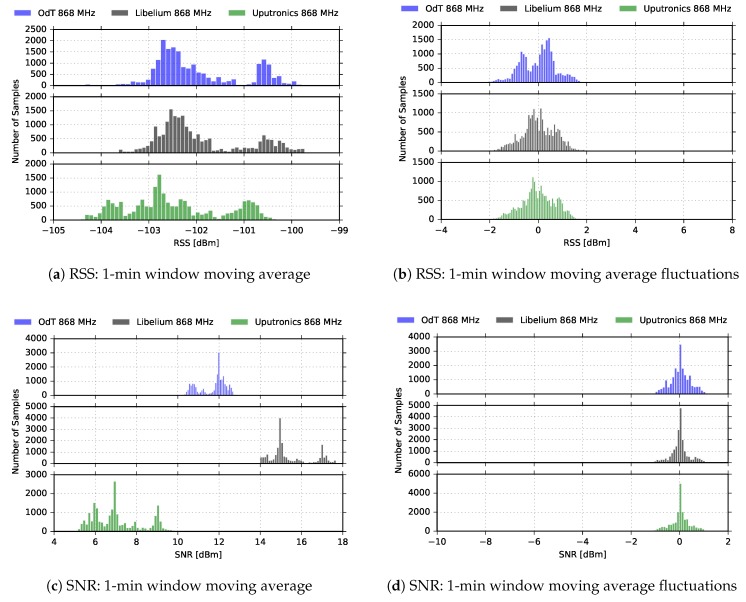
RSS and SNR histograms for ODT, Uputronics and Libelium: f=868MHz, BW=125kHz, SF=10, CR=4/6, LOS.

Figure 12 represents RSS and SNR histograms, based on the distribution of 1-min window moving average values, and differences between moving average values and current values for the same LoRa parameters. The distribution of the RSS amplitudes is obviously not Ricean, but it is expected since multipath propagation effect was present during only one interval of the experiment. Again, this has to be interpreted cautiously due to already mentioned reasons. The distribution for the difference between the 1-min window moving average values and current values can be approximated with Gaussian only in the SNR case, while for RSS bimodal distribution is observed. This can be explained with the presence of relatively pronounced multipath effects during one period of the experiment; however, further measurements are needed that would relate RSS bimodal distribution with multipath effects present in a limited interval within the experiment.

**Figure 13:**
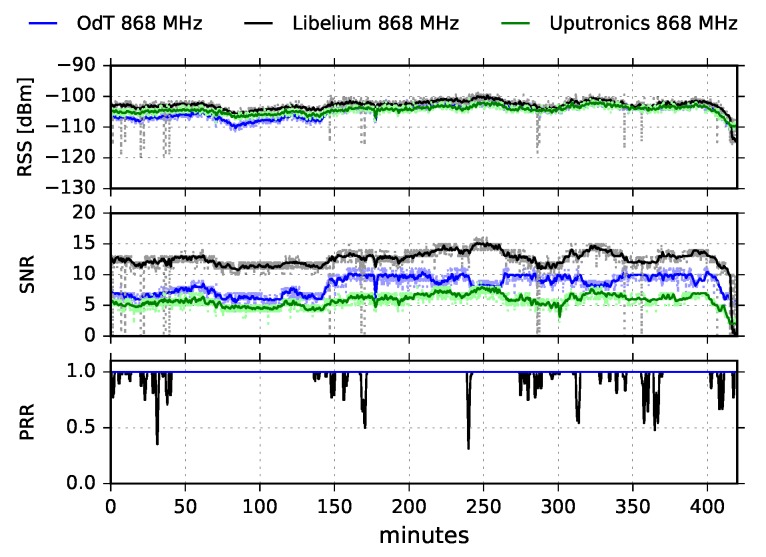
RSS, SNR, and PRR for ODT, Libelium, and Uputronics at 868MHz in LOS channel conditions for BW=125kHz, SF=12, and CR=4/6.

In Figure 13, results for BW=125kHz, SF=12, and CR=4/6are given. The small fluctuations of RSS for all three transceivers, approximately 2dB peak-to-peak, indicate that there was an unobtrusive multipath propagation, whose effects were not detrimental for PRR. On the other hand, sporadic packet loss that Libelium experienced is a consequence of short burst radio-interference, as can be seen on the plots. This claim is also additionally supported by the fact that moving averages of the RSS and SNR are significantly higher than the link margin during the whole measurement interval. We also observe that SNR follows RSS fluctuation trend for all three transceivers for most of the time, except for ODT: around the 250 and 325 min of the experiment we again notice that SNR decreases when RSS increases; a closer look reveals that RSS again reached −100dBm, replicating the same effects as in the previous experiment. Finally, by comparing the SNRs, it can be again noticed that ODT has the lowest noise floor and, consequently, the highest sensitivity.

**Figure 14:**
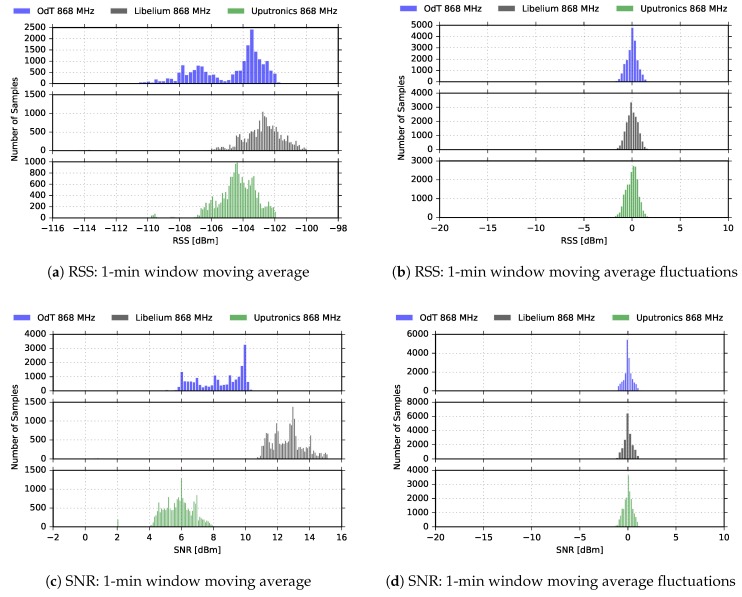
RSS and SNR histograms for ODT, Uputronics and Libelium: f=868MHz, BW=125kHz, SF=12, CR=4/6, LOS.

The histograms for the same experiment scenario, presented in Figure 14 confirms the reasoning applied in the previous experiments. Due to small-scale effects of multipath propagation, the distribution of the differences between RSS moving average values and current values is not bimodal; however, this has to be additionally confirmed with further experiments to be able to be generalized.

#### 4.1.2. ISM 434MHz

The experiments carried out at 434MHz were performed using a pair of ODT transceivers, exploring the same LoRa parameters as in the case for 868MHz band. In addition, the experimental setup was identical and deployed to operate simultaneously with the 868MHz setup.

**Figure 15:**
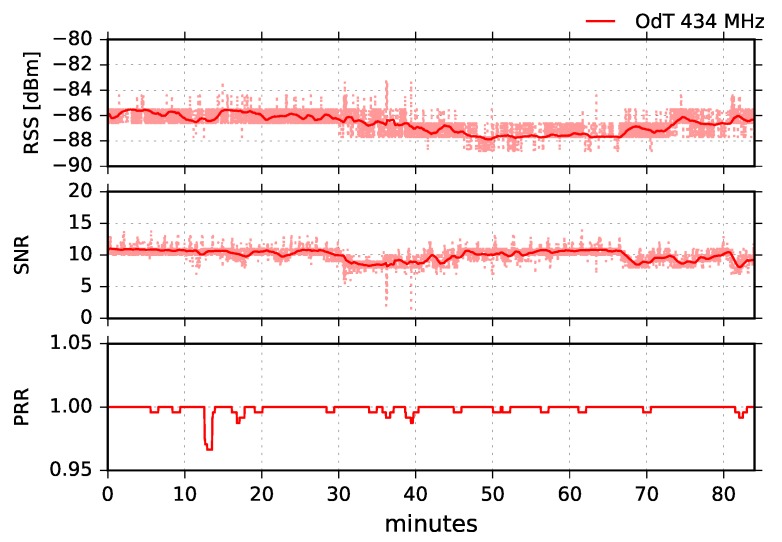
RSS, SNR, and PRR for ODT at 434MHz in LOS channel conditions for BW=125kHz, SF=7, and CR=4/6.

Figure 15 shows results for the following parameters: BW=125kHz, SF=7, and CR=4/6. It can be seen that the RSS is remarkably high during the whole measurement, the minimum value is −88dBm. Its moving average values fluctuate 3dB peak-to-peak indicating a small-scale multipath propagation. This also can be concluded if we compare it to the corresponding results at the 868MHz. Namely, the difference yields approximately 12dB. However, due to negligible terrain shielding in this case (see Table 2) and consequent free-space propagation, the difference should be 6dB, provided there are no multipath effects on propagation path. We can also observe a very small packet loss during the whole experiment which yields PL=0.15%.

The SNR fluctuations do not follow RSS fluctuation trend during the whole experiment. Referring to Figure 8, this can be easily explained. Namely, the SNR measurement chain is deeply in the saturation region, reflecting the oscillations that can be observed for RSS values lower than −100dBm.

**Figure 16:**
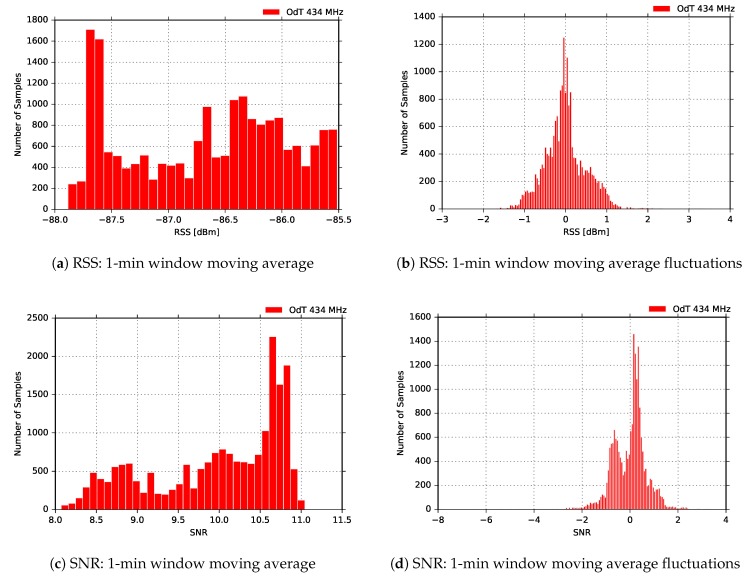
RSS and SNR histograms for ODT: f=434MHz, BW=125kHz, SF=7, CR=4/6, LOS.

Figure 16 shows the distribution of the moving average values of RSS and SNR calculated in the 1-min window, as well as distributions of the difference between their current and moving average values. The results shown are obtained using the following LoRa parameters: BW=125kHz, SF=7, and CR=4/6. We observe that difference between current and moving average values of RSS is nearly bimodal which is expected since small-to-medium-scale multipath effects can be observed. On the other hand, it can be noticed that for the same difference of SNR values there is a bimodal distribution, which may be explained by the deep saturation of the SNR measurement chain; however, this claim needs further investigation in order to be confirmed.

**Figure 17:**
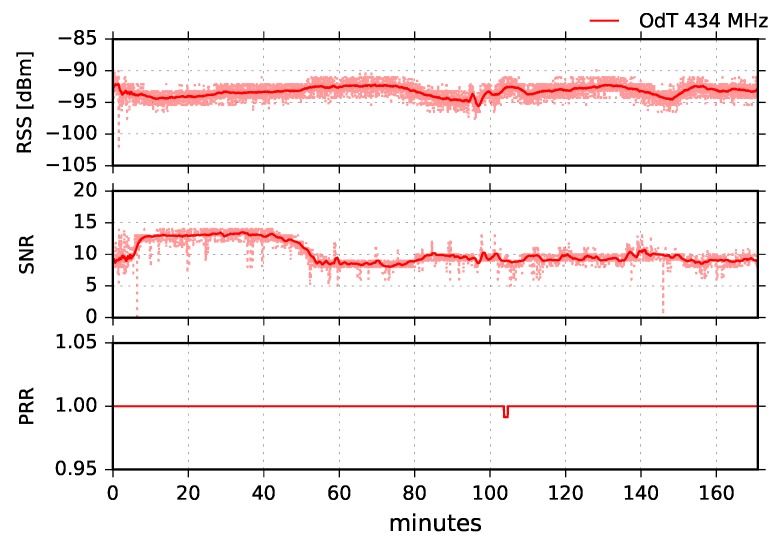
RSS, SNR, and PRR for ODT at 434MHz in LOS channel conditions for BW=125kHz, SF=10, and CR=4/6.

In Figure 17 results for the following LoRa parameters are given: BW=125kHz, SF=10, and CR=4/6. As in the previous experimental case, RSS fluctuation of moving average values are approximately 3dB peak-to-peak. The difference between the corresponding RSS values for the same LoRa parameters at 868MHz is slightly lower yielding 10dB. The same conclusion as in the case for SF=7 can be applied; this is also valid for SNR.

**Figure 18:**
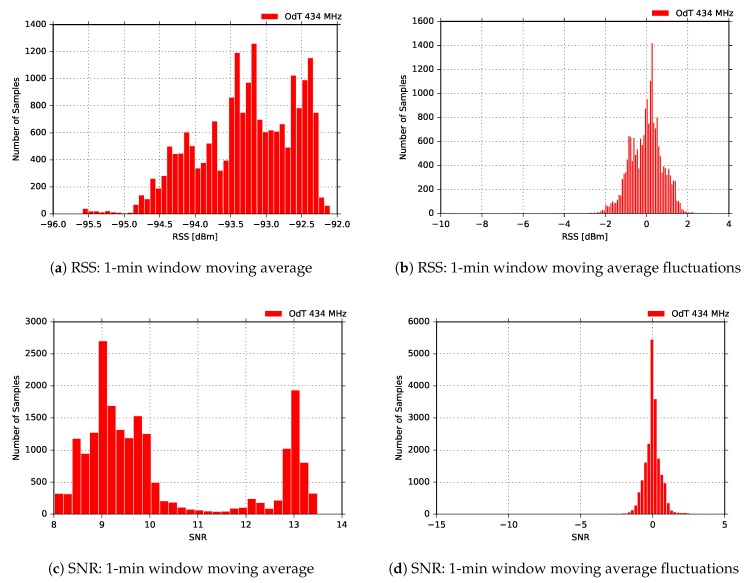
RSS and SNR histograms for ODT: f=434MHz, BW=125kHz, SF=10, CR=4/6, LOS.

Figure 18 shows RSS and SNR distribution of 1-min moving average and the difference between current values and moving average for the same LoRa parameters. Again, the small-scale multipath effects can be distinguished by inspecting the difference between current values and moving average values in Figure 18b. On the other hand, the moving average fluctuations in Figure 18d this time show Gaussian distribution, but the SNR measurement chain is on the onset of the saturation region, where the characteristic of the measurement chain is different than in the deep saturation region.

**Figure 19:**
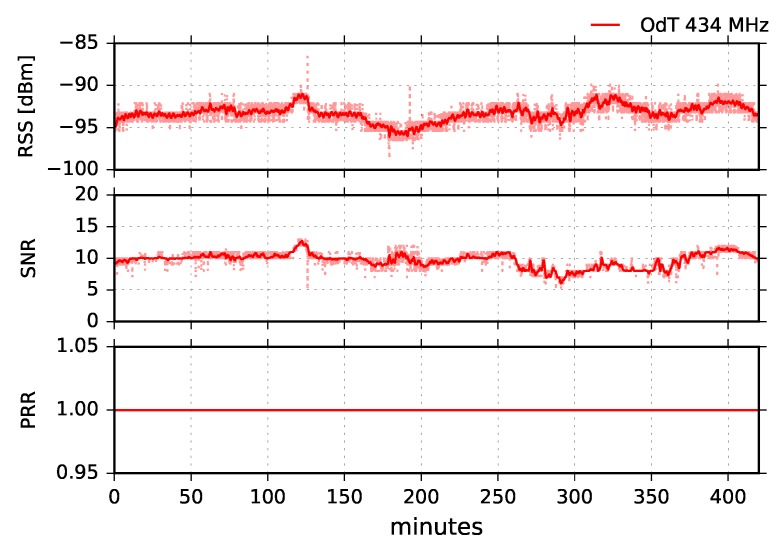
RSS, SNR, and PRR for ODT at 434MHz in LOS channel conditions for BW=125kHz, SF=12, and CR=4/6.

Finally, Figure 19 shows experimental results for the most sensitive mode of the ODT transceiver: BW=125kHz, SF=12, and CR=4/6. The RSS fluctuations in this mode are the highest, and multipath propagation is clearly observed. However, PRR is constantly PRR=1, which is expected due to highest sensitivity mode of operation. However, it should be noted that, counter-intuitively, these LoRa settings can also yield the highest packet loss due to very long packet air time (above 1 s) and increased susceptibility to interference. The uncorrelated fluctuations of SNR to RSS are due to the same reasons as in the other two experimental scenarios for 434MHz—the saturation of SNR measurement chain and oscillation which chain exhibits in that region.

**Figure 20:**
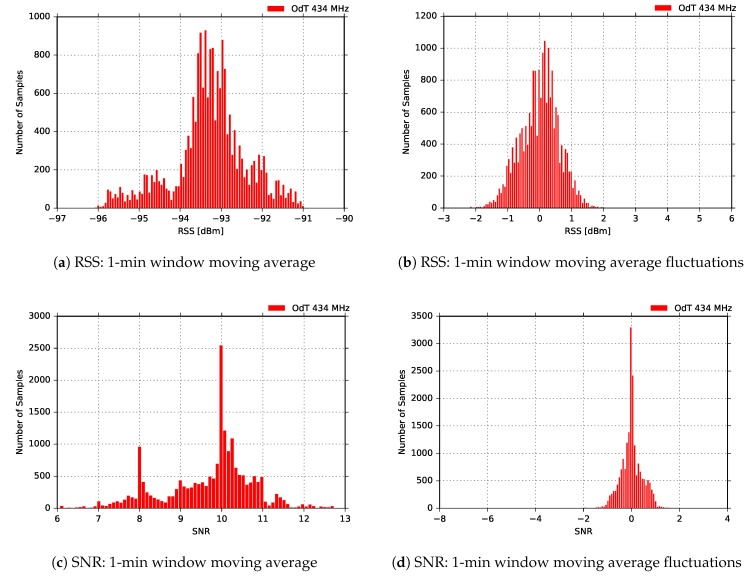
RSS and SNR histograms for ODT: f=434MHz, BW=125kHz, SF=12, CR=4/6, LOS.

Eventually, Figure 20 gives an insight in the distribution of the RSS and SNR moving averaged values, calculated on 1-min window for the same measurement. Here the same conclusions from the previous experimental case can be applied.

### 4.2. Obstructed LOS Scenario

This group of experiments had the aim of giving a deeper insight into LoRa links behavior over seawater when the LOS is completely blocked. We carried out experiments at both 868MHz and 434MHz using different settings and antennas. The trajectory was 28km long, see Figure 5.

**Figure 21:**
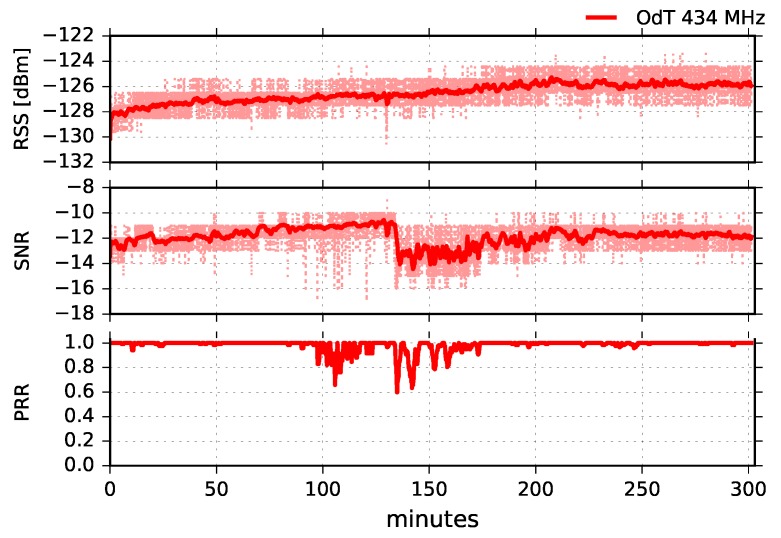
RSS, SNR, and PRR for ODT at 434MHz in obstructed path for BW=125kHz, SF=10, and CR=4/6.

Using low-cost off-the-shelf rubber-duck antennas, we were unable to establish a LoRa link on either frequency. Since we were on the boundary of the reception margin, we replaced the antennas on transmit side at both frequencies with wide-band three element collinear dipoles with higher gains: 8.5dBi at 868MHz, and 6.5dBi at 434MHz. The results of the experiment were the same as previously: there was no reception for any of the LoRa parameters. In the next step, we replaced the antennas at the receive side also with collinear dipoles at both frequencies of interest. Since this case is not suitable for most IoT commercial applications due to costly and bulky antennas at both sides, we tested only one set of LoRa parameters: BW=125kHz, SF=10, and CR=4/6. The results at 868MHz were the same as the previous ones: no reception. On the other hand, we had a reception for 434MHz, and the results are given in Figure 21.

Analyzing RSS, SNR, and PRR combined, it can be concluded that two different kinds of interferences affected transmission: (1) radio-interference, which caused main packet loss between 95 and 175 min of the experiment; and (2) interference due to multipath propagation which started after 140 min of the experiment. Radio-interference can be recognized by short dips in SNR while moving average values remain high. In the interval 140–175 min of the experiment, both radio-interference and interference caused by the multipath propagation affected transmission and PRR. This can be recognized due to short dips in SNR which reach link margin values, and by a drop in moving average values, respectively. Multipath propagation, in this case, could be caused by meteorological conditions and different refraction indexes of air masses between the transmit and receive side, as well as by the diffraction on knife-edge obstacle, made of natural rocks, which blocks LOS propagation (see Figure 5).

**Figure 22:**
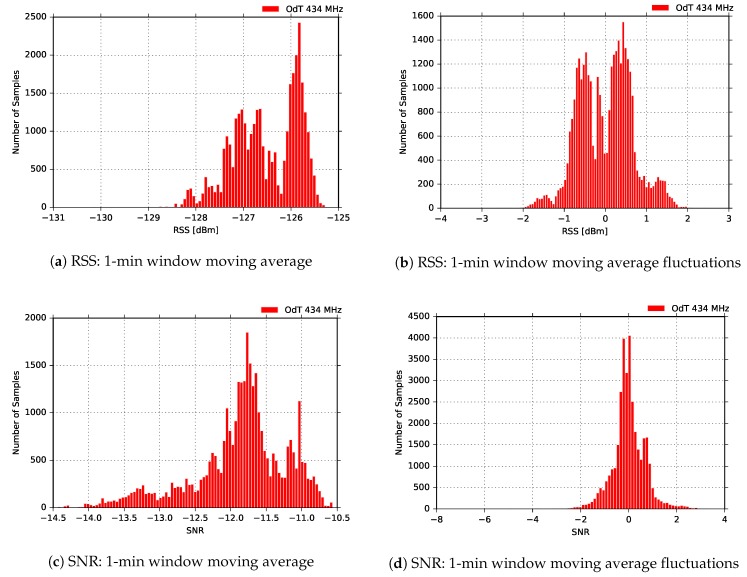
RSS and SNR histograms for ODT: f=434MHz, BW=125kHz, SF=10, CR=4/6, BLOS.

Figure 22 presents the distribution of RSS and SNR moving averages, and the difference between moving average values and their current values. The results are similar to all other results obtained for 434MHz band, except for the RSS fluctuations. Multimodal distribution (three modes) is observed in this case, which can indicate the presence of three intervals affected by different sources of interference combined; in order to confirm this claim, additional experiments need to be performed.

### 4.3. Discussion

The experimental results unambiguously show that LoRa links over seawater are feasible up to at least 22km for any combination of LoRa parameters in both frequency bands of interest, 868MHz and 434MHz if LOS condition between transmitter and receiver is guaranteed. Furthermore, the links are feasible using low-cost off-the-shelf rubber duck antennas which makes the results useful also in commercial deployments. Since there is a link margin of 15dB in 868MHz band, and approximately 20dB in 434MHz, it can be assumed that maximum achievable distance is significantly longer.

Moreover, the experiments also showed that there is a significant weather tolerance margin as well. Namely, it is well known that RSS decreases when ambient temperature increases [29]. This can impair signal reception if deployments are done in areas with high average temperature fluctuations during the year. Moreover, if the transceivers are mounted inside, e.g., IP-67 rated enclosure, the temperature can be even higher than ambient if the device is exposed directly to the sun [30]. Since our transceivers were deployed in unfavorable conditions in terms of weather conditions (they were mounted inside the IP-67 rated enclosure, directly exposed to the sun, with an ambient temperature of $28{\;}^{\circ }$C), it can be easily concluded that link feasibility can be extended for even higher ambient temperatures.

It is interesting to note that RSS fluctuations are smaller for a smaller spreading factor used. By increasing the spreading factor, the RSS fluctuations are increasing in amplitude in both frequency bands, resulting in a peak-to-peak value of 10dB for SF=12 at 868MHz. Additional investigation is needed to determine the cause, however, an explanation can be related to longer packet air times (due to higher SFs) and longer RSS measurement window which increases the probability for a different kind of interference to impact the measurement. Nevertheless, this did not cause a problem in the packet reception rate because the absolute value of RSS was substantially greater than the reception margin for the given LoRa parameters.

When the SNR measurement chains are not in the saturation region of operation, the values of SNR can be used to assess the link quality. It can be seen that ODT transceiver constantly has the highest SNR, as well as the highest PRR. This is the consequence of the lowest noise floor which is provided with a special hardware design approach described in [10]. This enables the ODT to decode very weak signals and maximize range.

The PRR is constantly high with negligible packet loss for all LoRa parameters tested, except for Libelium in the case of SF=7. This needs further investigation since RSS and SNR are considerably above the reception margin, and the other two receivers operated flawlessly for the same LoRa parameters. Besides that, in all other experimental scenarios packet loss is negligible, which implies that LoRa parameters can be chosen only according to the application specific requirements.

It is worth noting that in these experiments, compatibility between the only two manufacturers of LoRa ICs is validated since the transmitters were equipped with Semtech LoRa ICs, whereas one of the receivers was equipped with HopeRF LoRa IC. This practically means that there are no limitations in terms of hardware usage in different deployments.

The experiments also revealed that LoRa links can be established even in the presence of an obstacle that blocks the line of sight, provided that higher gain antennas are used at both ends. Nevertheless, there are certain limitations when it comes to their practical implementations since costly and bulky antennas are needed on both sides and transmission reliability is not guaranteed.

Results of the experiments imply that propagation over seawater does not impose any problem for LoRa transmission systems. The optimum set of parameters can be chosen considering only the particular application requirements since the reliable transmission can be achieved for virtually any combination of LoRa parameters within the bandwidth of BW=125kHz. Taking into account LoRa bit rate, these systems may be very useful for connection of environmental monitoring stations deployed in island countries. Moreover, this technology could be applied in early-warning systems for disaster prevention, for instance detecting volcano eruptions at long distances since the elevation of the volcano allows for a very long range LOS propagation paths.

## 5. Conclusions

The paper presented results acquired in the systematic measurement campaign which investigated LoRa transmission properties over seawater. A variety of hardware was deployed to operate in two different ISM bands, while different LoRa parameters were probed. The values reported by transceivers’ RSSIs were recalculated to correspond to the absolute values of the signal strength at the transceivers’ inputs, while the saturation threshold for SNR measurement chain was determined for each device. We were able to investigate short and long-term fluctuations of RSS, SNR, and PRR thanks to high sending rate and a huge number of packet sent in each of the experiments. Our main conclusions are: (1) RSSI of each transceiver in the test has a wide dynamic range, up to −50dBm, and exhibits linear behavior; (2) SNR measurement chain of each transceiver reaches saturation for RSS that are higher than −100dBm; (3) LoRa links over 22km long LOS propagation path over seawater are fully feasible for every combination of LoRa parameters, and in both ISM bands (868MHz and 434MHz); and (4) communication over 28km long obstructed LOS path is possible in 434MHz band, but using costly and bulky antennas. The significance of the results for LOS propagation path is particularly relevant due to the use of low-cost off-the-shelf rubber duck antennas.

## Figures and Tables

**Table 1:** Geographical details of the experimental sites.

Site	Latitude	Longitude	Elevation (above Sea Level)	Channel Condition	Link Length	Toponym, Country
A	45.703800	13.720100	56m	-	-	Miramare, Italy
C	45.521777	13.589979	133m	Line-of-Sight	22.6km	Pacug, Slovenia
D	45.477700	13.557300	64m	Obstructed path	28.2km	Kanegra, Croatia

**Table 2:** BotRf simulation results for 868MHz and 434MHz.

Parameter	Link AC: LOS Path (868,434)MHz	Link AD: Obstructed LOS Path (868,434)MHz
Free space path loss	(118.33,112.31)dB	-
Longley-Rice path loss	(118.26,112.25)dB	(176.70,166.12)dB
Attenuation due to terrain shielding	(0.07,0.06)dB	(56.47,51.91)dB
Mode of propagation	line-of-sight mode	double horizon, diffraction dominant

**Table 3:** Experiments’ technical details.

LoRa Parameters	Packet Air Time	Equiv. Bit Rate	Theoretical Sensitivity	Experiment Duration
SF7, BW125kHz, CR4/6	51.46ms	4557bps	−123dBm	84min
SF10, BW125kHz, CR4/6	313.34ms	813bps	−132dBm	171min
SF12, BW125kHz, CR4/6	1056.77ms	214bps	−137dBm	419min

## References

[1] Iridium Iridium Maritime Solutions. https://www.iridium.com/solutions/maritime/.

[2] Kazdaridis G., Symeonidis P., Zographopoulos I., Korakis T., Klun K., Kovac N. On the development of energy-efficient communications for marine monitoring deployments. Proceedings of the 2017 13th International Conference on Advanced Technologies, Systems and Services in Telecommunications (TELSIKS).

[3] Boydstun D., Farich M., McCarthy J., Rubinson S., Smith Z., Rekleitis I. Drifter sensor network for environmental monitoring. Proceedings of the 2015 12th Conference on Computer and Robot Vision.

[4] Pozzebon A., Cappelli I., Mecocci A., Bertoni D., Sarti G., Alquini F. (2018). A wireless sensor network for the real-time remote measurement of aeolian sand transport on sandy beaches and dunes. Sensors.

[5] Trasviña-Moreno C., Blasco R., Marco Á., Casas R., Trasviña-Castro A. (2017). Unmanned aerial vehicle based wireless sensor network for marine-coastal environment monitoring. Sensors.

[6] Semtech LoRa Modulation Basics. https://www.semtech.com/uploads/documents/an1200.22.pdf.

[7] Petajajarvi J., Mikhaylov K., Roivainen A., Hanninen T., Pettissalo M. On the coverage of lpwans: Range evaluation and channel attenuation model for lora technology. Proceedings of the 2015 14th International Conference on ITS Telecommunications (ITST).

[8] ETSI EN 300 220-1 V3.1.0 (2016-05). http://www.etsi.org/deliver/etsi_en/300200_300299/30022001/03.01.00_20/en_30022001v030100a.pdf.

[9] Li L., Ren J., Zhu Q. On the application of lora lpwan technology in sailing monitoring system. Proceedings of the 2017 13th Annual Conference on Wireless On-demand Network Systems and Services (WONS).

[10] Jovalekic N., Drndarevic V., Darby I., Zennaro M., Pietrosemoli E., Ricciato F. (2018). LoRa transceiver with improved characteristics. IEEE Wirel. Commun. Lett..

[11] Vangelista L. (2017). Frequency shift chirp modulation: The lora modulation. IEEE Signal Process. Lett..

[12] Reynders B., Pollin S. Chirp spread spectrum as a modulation technique for long range communication. Proceedings of the 2016 Symposium on Communications and Vehicular Technologies (SCVT).

[13] Spread SpectrumScene. The Advantages of Constant Envelope Modulation. http://sss-mag.com/cem.html.

[14] Bastille Networks Decoding LoRa, a Wireless Network for the Internet of Things. https://www.rsaconference.com/writable/presentations/file_upload/hta-f01-decoding-lora-a-wireless-network-for-the-internet-of-things_copy1.pdf.

[15] Petajajarvi J., Mikhaylov K., Pettissalo M., Janhunen J., Iinatti J. (2017). Performance of a low-power wide-area network based on LoRa technology: Doppler robustness, scalability, and coverage. Int. J. Distrib. Sens. Netw..

[16] Libelium Sx1272 LoRa Module for Arduino, Raspberry Pi and Intel Galileo—868 MHz [xbee socket]. https://www.cooking-hacks.com/documentation/tutorials/extreme-range-lora-sx1272-module-shield-arduino-raspberry-pi-intel-galileo.

[17] Uputronics Raspberry Pi+ LoRa Expansion Board. https://store.uputronics.com/index.php?route=product/product&product_id=68.

[18] Semtech Sx1276/77/78/79—137 MHz to 1020 MHz Low Power Long Range Transceiver. https://www.semtech.com/uploads/documents/DS_SX1276-7-8-9_W_APP_V5.pdf.

[19] Semtech Errata Note: Sx1276/77/78—137 to 1020 MHz Low Power Long Range Transceiver. https://www.semtech.com/uploads/documents/sx1276_77_78-errata.pdf.

[20] Silicon Labs EFM32GG990 Datasheet. https://www.silabs.com/documents/public/data-sheets/EFM32GG990.pdf.

[21] Micron Technology Inc. Nand128-a Nand256-a Datasheet. https://www.micron.com/~/media/documents/products/data-sheet/.../nandxxx-a.pdf.

[22] Semtech Sx1272/73—860 MHz to 1020 MHz Low Power Long Range Transceiver. https://www.semtech.com/uploads/documents/sx1272.pdf.

[23] Seeed Inc. Seeeduino Stalker v2.3. http://wiki.seeedstudio.com/Seeeduino_Stalker_v2.3/.

[24] HopeRF RFM95/96/97/98(w)—Low Power Long Range Transceiver Module. http://www.hoperf.com/upload/rf/RFM95_96_97_98W.pdf.

[25] Raspberry Pi Foundation Raspberry Pi3 Specifications. https://www.raspberrypi.org/products/raspberry-pi-3-model-b/.

[26] Zennaro M., Rainone M., Pietrosemoli E. (2017). Radio link planning made easy with a telegram bot. Smart Objects and Technologies for Social Good.

[27] Gaelens J., Van Torre P., Verhaevert J., Rogier H. (2017). Lora mobile-to-base-station channel characterization in the antarctic. Sensors.

[28] Youssef N., Wang C.X., Patzold M., Jaafar I., Tabbane S. On the statistical properties of generalized Rice multipath fading channels. Proceedings of the 2004 IEEE 59th Vehicular Technology Conference. VTC 2004-Spring.

[29] Boano C., Cattani M., Römer K. Impact of Temperature Variations on the Reliability of LoRa—An Experimental Evaluation. Proceedings of the 7th International Conference on Sensor Networks—Volume 1 (SENSORNETS).

[30] Boano C.A., Brown J., He Z., Roedig U., Voigt T. Low-power radio communication in industrial outdoor deployments: The impact of weather conditions and ATEX-compliance. Proceedings of the 1st International Conference on Sensor Networks Applications, Experimentation and Logistics (SENSAPPEAL).

